# Multiple congenital anomalies and adverse developmental outcomes are associated with neonatal intensive care admission and unilateral hearing loss

**DOI:** 10.3389/fped.2022.1068884

**Published:** 2023-01-10

**Authors:** Lucy M. Horrocks, Pádraig T. Kitterick, Dulip S. Jayasinghe, Karen R. Willis, Katherine R. M. Martin, Abhijit Dixit, Sally K. Thornton

**Affiliations:** ^1^ Hearing Sciences, School of Medicine, The University of Nottingham, Nottingham, United Kingdom; ^2^ NIHR Nottingham Biomedical Research Centre, Nottingham University Hospitals NHS Trust, Nottingham, United Kingdom; ^3^ Neonatal Intensive Care Unit, City Hospital Campus, Nottingham University Hospitals NHS Trust, Nottingham, United Kingdom; ^4^ Children’s Audiology, Nottingham University Hospitals NHS Trust, Nottingham, United Kingdom; ^5^ Children’s Development Centre, City Hospital Campus, Nottingham University Hospitals NHS Trust, Nottingham, United Kingdom; ^6^ Clinical Genetics, City Hospital Campus, Nottingham University Hospitals NHS Trust, Nottingham, United Kingdom

**Keywords:** unilateral hearing loss, neonatal intensive care, congenital anomalies, developmental outcomes, congenital hearing loss, paediatrics

## Abstract

**Aim:**

To determine congenital and developmental outcomes of children with Unilateral Hearing Loss (UHL) who were admitted to the Neonatal Intensive Care Unit (NICU).

**Method:**

Retrospective, single-site study that followed 25 children with permanent congenital UHL *and* a NICU admission to a NICU of Nottingham University Hospital. Birth and two-year developmental follow-up data were collected. They were compared to matched control group who had a NICU admission but no hearing loss (matched on gestational age, weight and sex).

**Results:**

The median birthweights, gestational ages and number of days spent on the NICU for the UHL population were 2510 g, 36 weeks, and 12 days respectively. Most children (20/25; 80%) with UHL *and* a NICU admission were diagnosed with a congenital anomaly within the first two years of life. Only half (13/25) of these children were diagnosed with a congenital anomaly at discharge. Children with UHL *and* a NICU admission were more likely than the matched group (NICU admission only; *p* < .001) to have multiple congenital anomalies. We found a positive association between multiple congenital anomalies and developmental impairment for the NICU graduates with UHL (*p* = .019). This UHL-NICU group were also more likely than the matched NICU children to have developmental impairment (7/25 vs. 0/25; *p* = .01), speech and language therapy (13/25 vs. 1/25; *p* < .001), inner ear malformations (14/25 vs. 0/25, *p* < .001) or craniofacial anomalies (12/25 vs. 2/25; *p* = .004).

**Interpretation:**

Children with UHL *and* a NICU admission were at high risk of congenital anomalies and certain adverse developmental outcomes. Improved congenital anomaly screening is needed at birth for this population. Having multiple congenital anomalies suggests closer developmental monitoring is needed. This study contributes towards producing clinical screening and management guidelines to ensure consistent high-quality care for this unique population.

## Introduction

Babies born with unilateral hearing loss (UHL) who were admitted to the neonatal intensive care unit (NICU) are a very understudied population, with *no* current literature on their specific developmental outcomes. Currently there are *no* national hearing or NICU guidelines surrounding the management of this population or of the birth population of children with UHL. UHL is hearing loss in only one ear, it's prevalence in the birth population is estimated at 0.3–1 per 1,000 births (1–3) and whilst there is little literature documenting it's prevalence in the NICU population, some studies estimate 1.2%–4.6% (4, 5).

There has been no research investigating two-year outcomes of children with UHL *and* a NICU admission, although there has been research into the birth population of children with UHL and for children with normal hearing with a NICU admission.

Approximately 40% of children with UHL require speech and language therapy (SLT) (6). A need for further academic support has also been identified in this population, with studies finding that 45% of children with UHL need an Individualised Education Program (IEP) (7) and almost a third of children with UHL fail a grade (8). It is established in the literature that congenital anomalies such as craniofacial abnormalities and inner ear malformations are common in this population (3, 9–11) and the prevalence of bony malformations in the inner ear and/or internal auditory canal was markedly higher in infants with congenital UHL than in infants with bilateral hearing loss (9), with about two thirds (66.7%) of children with UHL having inner-ear and/or internal auditory canal malformations (12). However, little research has been undertaken into the number and further “non ear” related variety of congenital anomalies present in children with UHL.

For children admitted to the NICU, Schiariti et al. found that 12.6% of term babies had a congenital anomaly; 3.1% of them were cardiac and/or circulatory anomalies, 2.7% were gastrointestinal anomalies and 0.4% were face and neck anomalies (13). Those that are preterm and/or have a low birth weight are more likely to have visual and auditory impairments (14), with 3%–5% of babies <1500 g having a hearing impairment, compared to less than 1% of babies born at term (15). Babies born pre-term and/or of low birth weight are more likely to have learning difficulties (14). One study found that a third of preterm babies (32–36 weeks) had motor, speech and educational difficulties in childhood (14).

The absence of published literature surrounding the developmental outcomes of children with UHL *and* a NICU admission has highlighted the need for this exploratory study. The primary aim is to describe the congenital, anatomical and behavioural outcomes of this population, with the aim to determine a constellation of features specifically associated with this group of children. Genetic screening is not currently funded for children with UHL in the UK.

This information from a larger cohort could inform the development of clinical guidelines on the follow up and management of this understudied population, ensuring effective and consistent care.

## Materials and methods

### Participants

This retrospective, longitudinal case-controlled cohort study documents routinely collected NICU discharge data, two-year developmental follow-up data and hearing aid treatment data which was collected and analysed for patients with UHL and without UHL *and* a NICU admission to Nottingham University Hospitals. For the UHL infant group the inclusion criteria included a NICU admission and a referral from the UK National Newborn Hearing Screening Programme (NHSP) with a subsequent diagnosis of permanent UHL confirmed *via* auditory brainstem response (ABR) for birth dates between February 28, 2008, and 12 July 2019. This gave the final cohort of 25 patients (13 females, 12 males; median gestational age 36 weeks; median birthweight 2510 g).

Data was also collected for matched and peer controls. The matched control patients had passed the NHSP screening and were matched on sex, birthweight (±10 g), gestational age (±1 week) and whether they spent ≥48 h NICU, as it is already well established that these variables impact a baby's development. Few exceptions were made when no matched babies fit these criteria; the weight range was increased by 10 g, and one patient was not matched on sex. If more than one baby fit the matched control criteria, then the baby born most recently was chosen. Peer control patients had passed the NHSP screening and were matched on date of birth (±4 days) and sex. Their weights, gestational ages and days spent on the NICU were recorded. No significant difference was detected regarding the birthweights, gestational ages and number of days spent on the NICU between the children with UHL and their matched controls or peer controls (*p* > 0.05 for all comparisons).

The NEAT database has been approved by Research Ethics Committee (REC 22/SC/0337; IRAS 292263).

### Procedure

Data recorded between 0 and 2 years included data from (pre-term) birth to 2 years. Data recorded at birth was taken from NICU discharge notes. We analysed all data from birth to the time of the study and utilised paper notes or NHS databases. Demographic data, discharge summaries and the patient's first postcode were recorded from the neonatal database, Badger. If a patient had multiple anomalies within one organ or organ system, this was still recorded as one anomaly.

Developmental impairment is a diagnosis of their development which is not in the normal range for their corrected gestational age. Specifically, a child was diagnosed with developmental impairment if their developmental skills fell two SD or more below the population mean in two or more developmental domains. Developmental impairment was included in this study if it was stated in the patient notes that they had a global developmental delay, fine motor delay, delay in communication, developmental impairment or had been referred for developmental needs.

In terms of eligibility for SLT in the study area, the local SLT specialist hearing impaired team only accept referrals for children with severe/profound bilateral hearing loss and auditory spectrum neuropathy disorder. Referral to local community SLT is possible for children with other degrees of hearing loss, however this is not done as routine at point of diagnosis by the current service.

Home postcode on discharge from NICU was used to determine deprivation index (16, 17). The lower the index of multiple deprivation decile, the more deprived the area was that the patient lived [1 = most deprived, 10 = least deprived (16, 17)]. These patients were then split into two groups – (i) index of multiple deprivation decile of <5 (more deprived) and (ii) index of multiple deprivation decile of ≥5 (less deprived).

Follow-up data for the UHL cases and their matched counterparts were collected and used to determine abnormal anatomical features between 0 and 2 years. There were four exceptions in the UHL cohort; three eye problems and one inner ear malformation were detected *after* 2 years. A consultant neonatologist determined which anomalies were congenital.

The date of first fitting of a hearing device and the types of devices trialled were recorded. It was then calculated how many patients had their device fitted before 1 year of age. Patients not recorded on the database were assumed to have never trialled a hearing device.

Hearing thresholds for the patients with UHL were recorded and their most recent audiology report was used. The patients with UHL were then split into two groups, those that had mild/moderate hearing loss and those that had severe/profound hearing loss according to the British Society of Audiology guidelines (18).

See Supplementary Appendix S2 for the full list of diagnoses included and definition of major and minor craniofacial abnormalities.

Missing data: The number of children with UHL may be higher than we recorded in this population as cases may be missed for a number of reasons: Firstly, while many US screening programmes include babies with permanent mild BHL and UHL in their target group, NHSP in the UK does not. It aims to identify all children with a moderate-profound permanent HL in the better hearing ear. As a by-product, the screen will identify babies who have UHL and, in some cases, mild permanent hearing loss, as well as temporary hearing loss. Babies with UHL may also be missed if they had moved out of area after NICU admission or if their data was not available on Badgernet.

### Statistical analysis

A power calculation was not performed as the sample size was limited by the number of available UHL cases with NICU admissions registered at the Nottingham University Hospitals, 2008–2019. Mann–Whitney *U* test was performed for non-categorical data and chi-squared for categorical data (or Fischer's Exact if f *n* ≤ 5 in either group being compared).

Shapiro-Wilk test indicated that the non-categorical data were not normally distributed.

## Results

In this study, 14,538 babies were admitted to the NICU, 25 of whom had UHL, making the prevalence of UHL in this NICU cohort approximately 0.17%. This is lower than previous reports (1.23%–4.6%) (4, 5).

### Congenital anomalies

Congenital anomalies that were detected at birth, and between discharge and two years, were documented in Table 1. Over half (13/52) of cases with UHL were diagnosed with a congenital anomaly at birth, with the majority of cases being diagnosed with multiple congenital anomalies (8/13). Surprisingly, analyses reveal that more than a quarter (*n* = 7) of patients with UHL were diagnosed with an anomaly that was thought to be congenital, post-discharge, but within the first 2 years of life. Therefore, when the total number of children with UHL with congenital anomalies detected at birth and post-discharge are combined, the majority (20/25) of babies with UHL had a least one congenital anomaly. Most (16/20) of these cases had multiple congenital anomalies. Children with UHL were significantly more likely to have a congenital anomaly that was diagnosed either at birth (*p* < 0.001) or post NICU discharge between 0 and 2 years (*p* < 0.001) compared to their matched counterparts. They were also significantly more likely to have multiple congenital anomalies (>1 anomaly) at birth (*p* = 0.004) and between 0 and 2 years (*p* < 0.001) compared with the matched control group.

**Table 1:** Congenital anomalies detected at birth.

	At birth	Between birth and 2 Years
Number of congenital anomalies	UHL (*N* = 25)	Matched controls	UHL (*N* = 25)	Matched controls (*N* = 25)
0^a^	12 (48%)	24 (96%)	5 (20%)	21 (84%)
≥1^a^	13 (52%)	1 (4%)	20 (80%)	4 (16%)
≥2^a^	8 (32%)	0 (0%)	16 (64%)	2 (8%)
≥3	4 (16%)	0 (0%)	13 (52%)	2 (8%)
≥4	2 (8%)	0 (0%)	8 (32%)	0 (0%)

^a^ Indicates there was a statistically significant difference between the cases (UHL) and matched controls using a chi-squared test (*n* > 5), or Fischer's Exact where *n* ≤ 5 (*p* < .05).

### Abnormal anatomical features

Table 2 presents the abnormal anatomical features in the UHL cohort between 0 and 2 years. A statistically significant difference was found regarding total number of anomalies between children with UHL (71) and their matched controls (21) (*p* < 0.001). Children with UHL were 6 times more likely to have vision and eye issues, 5 times as likely to have other malformations, 4 times as likely to have neurological and spinal issues, 3.5 times as likely to have gastrointestinal problems, 3 times as likely to have renal anomalies and 2.5 times as likely to have cardiac abnormalities than their matched counterparts. However, these differences were not found to be statistically significant (*p* > 0.05). Only the differences in inner ear malformations (IEM) and craniofacial anomalies were statistically significant, yielding a *p*-value of <0.001 and 0.004 respectively. Division of craniofacial anomalies into subgroups revealed a significant difference from matched controls only detected for ear anomalies (*p* < .05). No statistical differences were found between the patients with UHL and their matched controls for respiratory, metabolic or neuromotor anomalies.

**Table 2:** Describing abnormal anatomical features detected between birth and 2 years.

	Detected between birth and 2 years		Detected between birth and current age	
Anatomical anomaly	UHL (*N* = 25)	Matched controls (*N* = 25)	UHL (*N* = 25)	Matched controls (*N* = 25)
Inner ear malformations	14 (56%)	0 (0%)	14 (56%)^a^	0 (0%)
Craniofacial anomalies	12 (48%)	2 (8%)	12 (48%)^a^	2 (8%)
Major anomaly only	4 (16%)	0 (0%)	4 (16%)	0 (0%)
Minor anomaly only	6 (24%)	2 (8%)	6 (24%)	2 (8%)
Major and minor anomaly	2 (8%)	0 (0%)	2 (8%)	0 (0%)
Ear anomaly only	5 (20%)	0 (0%)	5 (20%)^a^	0 (0%)
Face or head anomaly only	4 (16%)	2 (8%)	4 (16%)	2 (8%)
Ear and face or head anomaly	3 (12%)	0 (0%)	3 (12%)	0 (0%)
Neurological and spinal	5 (20%)	1 (4%)	8 (32%)	2 (8%)
Gastrointestinal	5 (20%)	1 (4%)	7 (28%)	2 (8%)
Vision and eye	1 (4%)	0 (0%)	6 (24%)	1 (4%)
Other malformations	5 (20%)	1 (4%)	5 (20%)	1 (4%)
Cardiac	5 (20%)	0 (0%)	5 (20%)	2 (8%)
Respiratory	3 (12%)	0 (0%)	4 (16%)	3 (12%)
Metabolic	2 (8%)	0 (0%)	4 (16%)	5 (20%)
Renal	1 (4%)	1 (4%)	3 (12%)	1 (4%)
Neuromotor	1 (4%)	0 (0%)	3 (12%)	2 (8%)
Total number of anomalies	41	6	71^b^	21

^a^ Indicates there was a statistically significant difference between the cases (UHL) and matched controls using Fischer's Exact (as *n* ≤ 5).

^b^ Indicates there was a statistically significant difference between the cases (UHL) and matched controls using Mann–Whitney *U* test.

### Behavioural outcomes

Table 3 describes the behavioural outcomes of the UHL patients that were recorded to date. 52% of patients with UHL had SLT, 28% had a developmental impairment and 16% had a learning disability. There was a significant difference between the UHL group and their matched controls for SLT (*p* < 0.001) and developmental impairment (*p* = .01). It was observed that *all* of the UHL patients with a developmental impairment also had multiple congenital anomalies, with 4/7 (57%) having ≥4 congenital anomalies. Analyses revealed that patients with UHL *and* a developmental impairment were statistically more likely to have multiple congenital anomalies than those without a developmental impairment (*p* = .019).

**Table 3:** Behavioural outcomes recorded to date.

	UHL (*N* = 25)	Matched controls (*N* = 25)
Speech and language therapy^a^	13 (52%)	1 (4%)
Developmental impairment^a^	7 (28%)	0 (0%)
Learning disability	4 (16%)	0 (0%)
Autism	1 (4%)	0 (0%)

^a^ Indicates there was a statistically significant difference between the cases (UHL) and matched controls using Fischer's Exact (as *n* ≤ 5).

### Syndromic and genetic data

Genetic testing was carried out on twelve patients with UHL, and eleven results were obtained. Four of these patients had a genetic variant, two of which were pathogenic and two of uncertain significance. It is not known if these variants were causative of hearing loss. Three out of the four patients that had a genetic variant also had a phenotypic syndrome diagnosed by a paediatrician.

Six patients with UHL were diagnosed with a syndrome, four of which are known to be associated with hearing loss. These were Oculo-auriculo-vertebral Syndrome (19), Large Vestibular Aqueduct Syndrome (20), Klippel Feil Syndrome (21) and Beckwith Wiedemann Syndrome (22). Of those with a syndromic diagnosis, all 6 had multiple congenital anomalies, 4 had a developmental impairment and 4 were enrolled in SLT.

No syndromic or genetic data were recorded for the matched controls.

### Hearing data

Fourteen of the 25 patients with UHL had mild or moderate hearing loss and 11 had severe or profound hearing loss in their affected ear. More than half of the patients (14/25) had an index of multiple deprivation decile of <5 (associated with lower socio-economic status according to their postcode) on discharge from the NICU. Nearly half (12/25) had trialled a hearing device, and most of these patients (10/25) still used a hearing device at the time of the study. The patient's degree of hearing loss or deprivation decile did not significantly affect whether they had a hearing device, their age of fitting of the device or what device they used. Most patients with UHL that were fitted with a hearing device were fitted after their first birthday (8/12;67%) irrespective of their degree of hearing loss or deprivation, with an average age of first fitting of 3 years 1 month. Patients with UHL who had SLT or developmental impairment were significantly more likely to have trialled a hearing device than those that did not (*p* = .047 and *p* = 0.019 respectively). Five of the seven children with UHL and a developmental impairment also received SLT.

## Discussion

Children with UHL *and* a NICU admission were at high risk of multiple congenital anomalies and certain adverse developmental outcomes. Targeted clinical screening—genetic and clinical follow-up is needed at birth for this discrete population.

These data indicate that children with UHL *and* a NICU admission were more likely than their matched counterparts to have congenital anomalies, developmental impairment and SLT. Approximately two thirds (64%) of patients with UHL had multiple congenital anomalies but not all congenital anomalies were detected at discharge (7/25, detected post-discharge). This information suggests better screening for congenital anomalies would be advantageous in this population. This would be particularly beneficial as a congenital anomaly was found to be positively associated with a developmental impairment and could be used as an indicator for closer developmental follow up in early life.

The prevalence of UHL and NICU admission was 0.17%—lower than previous reports (1.23%–4.6%) (4, 5). Varied methodology could explain this difference, for example babies on the NICU for ≤5 days were excluded in one of these studies. Any baby with suspected sepsis is routinely admitted to the NICU in Nottingham University Hospitals which differs from other regions. Another possibility is despite universal NHSP, UHL is under-reported or undetected in this cohort.

### Congenital anomalies

In this study, half (14) of UHL cases were diagnosed with a congenital anomaly at birth. A further quarter (7) patients had congenital anomalies that weren't detected at birth but were detected within the 2 years following. This prevalence is higher than the 29% of congenital anomalies recorded in the literature for the general population of babies with UHL (3); this suggests that NICU-UHL is a red flag for anomalies that don't come to light until post-discharge. This population of babies with UHL and NICU admission were also more likely than their matched counterparts (normal hearing and NICU admission) to have multiple congenital anomalies (64% vs. 8%), further highlighting the importance of screening and detection for NICU-UHL babies.

### Abnormal anatomical features

This study supports the already published literature that IEM and craniofacial anomalies are positively associated with UHL and also suggests that NICU admission doesn't increase the likelihood of having these conditions within the UHL cohort, as the prevalence is similar to the UHL well-baby population (3, 9, 10).

Yelverton et al. (2013) showed that 2.4% of babies with UHL (combined well and NICU cohort patients) also had a gastrointestinal problem detected at birth (3), which is 10× lower than the 28% of patients (UHL-NICU) who had a gastrointestinal problem recorded in this study. A NICU admission and diagnosis of UHL could be strongly associated with gastrointestinal problems, or it may be that gastrointestinal issues develop over time. It is possible that we had a lower detection threshold for documenting gastrointestinal problems than Yelverton. Patients with UHL were more likely to have a gastrointestinal problem than their matched counterparts (7/25 vs. 2/25), and most of the associated gastro-intestinal problems (5/7) were detected at birth. Further research needs to be conducted into this association.

In Table 2 it is apparent that one third, (32%) of the UHL cases were identified as having neurological and spinal issues and a quarter (24%) had vision and eye problems by the age of two. Again we detected a much larger prevalence than reported by Yelverton et al.'s birth population UHL study (4.3%) (3). These data indicate that admission to the NICU and having UHL presents a higher cumulative risk of neurological and/or eye issues. Furthermore, many of these problems may develop after birth; 38% (3/8) of those with spinal and neurological issues and 83% (5/6) of those with vision and eye problems in this cohort developed them in early childhood. This particularly high prevalence of vision and eye problems, 6 times greater than their matched counterparts, suggests a need for closer ophthalmic follow up in this population with UHL.

20% of patients with UHL *and* a NICU admission were found to have cardiac anomalies between 0 and 2 years, which lies between the two values for the general UHL population (41%) and 12% for babies who also have a co-existing JCIH – US risk factor (3) these are all higher than the 8% of matched controls. Many cardiac anomalies that were documented (for example, heart murmur, atrioventricular septal defect), were discounted as they are extremely common in the general NICU population as they are usually not significant, result from prematurity and often resolve with age. Methodological considerations (what is counted as a cardiac anomaly) may account for some of the differences between our data and the current literature.

### Behavioural outcomes

This study identified a prevalence of 52% of UHL cases needing SLT, 13 times greater than their matched controls, but in keeping with the majority of the current literature for well babies with UHL and no NICU admission (6, 7). This suggests it is the UHL and not the other underlying health conditions associated with admission to the NICU which increases the likelihood of needing SLT for patients with UHL. This again highlights the need to target NICU graduates with UHL for referral and follow-up.

It was interesting that over a quarter of the UHL cases had a developmental impairment, which was significantly more than their matched controls (28% UHL vs. 0% controls). There is little literature surrounding the association between UHL and developmental impairments. One study found one fifth of children with UHL (both well and NICU populations) were diagnosed with developmental delay (23), which is similar to the findings of this study (28%). Specific developmental follow-up for patients with UHL could help to identify developmental impairment earlier and provide earlier interventions, which could lead to better outcomes and quality of life. Furthermore, all UHL cases in this study with developmental impairment had multiple congenital anomalies, suggesting that having multiple congenital anomalies could be an indicator for closer developmental monitoring in this population throughout early childhood.

### Syndromic and genetic outcomes

In this study, nearly one quarter (6/25) of the UHL cases were diagnosed with a recognised syndrome, 4 of which were found to be associated with hearing loss. It is interesting that these were not the syndromes most associated with UHL (for example, Waardenburg Syndrome), suggesting that perhaps there is a different subset of syndromes yet to be identified, that are more likely to be associated with UHL *and* NICU admission. One study found that 1 in 110 patients (0.9%) with UHL had a syndrome that was associated with hearing loss, which is much lower than the 16% found in this study (3). This suggests that infants with a syndrome and UHL are more likely to also have a NICU admission.

Studies have revealed the percentage of UHL associate with a family history is approximately 3.7%–13% (9, 24, 25), which is similar to the number of UHL cases with genetic variants in this study (16%). There is little to no research on the specific genetic variants associated with UHL. Furthermore, three of the four patients with a genetic variant also had a diagnosed syndrome, suggesting that the syndromes may be linked to specific genetic variants. A national study investigating genetics and UHL cases would need to be conducted to confirm this. Currently genetic screening is not recommended or funded for infants diagnosed with UHL in the UK.

### Hearing outcomes

In this study, 40% of patients with UHL were currently using a hearing device, which is similar to pre-existing literature (6). Patients with UHL that received SLT were significantly more likely to have trialled a hearing device than those that had not received SLT. A study into children with bilateral hearing loss (BHL) by Tomblin et al. (2015) identified that hearing aids can improve language outcomes over time in these children (26). There is sparse literature available to indicate that children with UHL that are struggling with their speech and language development may benefit more from a hearing device or a trial of a device in their early years. There is some evidence to suggest that wearing a hearing device can improve quality of life, especially in those suffering with speech and language or academic and behavioural issues, whereas other studies have found that hearing devices may not be beneficial for younger children with UHL and do not improve speech recognition (27–30). Cochlear implantation is not currently funded for children with UHL in the UK, recent preliminary studies have shown improvement in some areas for children with UHL following implantation (31). More research is needed in this area. Furthermore, the majority (6/7) of patients with UHL that had a developmental impairment had trialled a hearing device, which may be due to the positive association between developmental delay and SLT. Furthermore, as there are no current UK NHS guidelines for the management of UHL in children, audiologists may use SLT or diagnosis of a developmental impairment to guide them on management with a device, as well as use this information to suggest to parents that their child trial a device. However, by the time the child needs SLT this may be too late; earlier device trials could be important during the critical period for language acquisition. Parents may be more willing to trial a device if they see the developmental effects UHL has on their child. Out of the home, eg in nursery and playgroups it could be vital for children with UHL (particularly NICU graduates) to use a hearing device and employ a remote microphone system as it is known that deciphering speech in noise is particularly difficult for people with UHL. Currently there is not consistent funding for remote microphone systems and SLT for children with UHL.

### Future work

Not all patients with UHL *and* a NICU admission go onto develop congenital anomalies or abnormal anatomical or behavioural outcomes; further research is required into why this is the case and are their neuroprotective factors which help pre or postnatally (eg maternal magnesium or prenatal steroids). It is possible that there is a subset of patients in this cohort that have certain risk factors that increase their chance of having adverse developmental outcomes. For example, further research into the association between multiple congenital anomalies and developmental outcomes could be conducted.

## Conclusion

Research into the developmental outcomes of patients with UHL has mainly focused on the general population, not the cohort that has also been admitted to the NICU. This longitudinal study identified many adverse outcomes in this unique population, which is a step towards identifying a constellation of features associated with UHL in babies who have been admitted to the NICU. This study can contribute towards developing guidelines surrounding the screening, follow up and management of these patients, which would benefit both clinicians and patients.

## Data Availability

The data analyzed in this study is subject to the following licenses/restrictions: Currently datasets are not available to researchers outside of NUH Nottingham. Datasets available on application to NEAT database team. Requests to access these datasets should be directed to dulip.jayasinghe@nuh.nhs.uk.

## References

[1] Berninger E Westling B. Outcome of a universal newborn hearing-screening programme based on multiple transient-evoked otoacoustic emissions and clinical brainstem response audiometry. Acta Otolaryngol. (2011) 131(7):728–39. 10.3109/00016489.2011.554440 21466262

[2] Prieve B Dalzall L Berg A Bradley M Cacace A Campbell D The New York State universal newborn hearing screening demonstration project: outpatient outcome measures. Ear Hear. (2000) 21:104–17. 10.1097/00003446-200004000-00005 10777018

[3] Yelverton JC Dominguez LM Chapman DA Wang S Pandya A Dodson KM. Risk factors associated with unilateral hearing loss. JAMA Otolaryngol Head Neck Surg. (2013) 139(1):59–63. 10.1001/jamaoto.2013.1097 23329092

[4] Chang J Oh SH Park SK. Comparison of newborn hearing screening results between well babies and neonates admitted to the neonatal intensive care unit for more than 5 days: analysis based on the national database in Korea for 9 years. PLoS One. (2020) 15(6):e0235019. 10.1371/journal.pone.0235019 32559227 PMC7304604

[5] Stadio AD Molini E Gambacorta V Giommetti G Volpe AD Ralli M Sensorineural hearing loss in newborns hospitalized in neonatal intensive care unit: an observational study. Int Tinnitus J. (2019) 23(1):31–6. 10.5935/0946-5448.20190006 31469525

[6] Lieu JE Tye-Murray N Fu Q. Longitudinal study of children with unilateral hearing loss. Laryngoscope. (2012) 122(9):2088–95. 10.1002/lary.23454 22865630 PMC3467198

[7] Rachakonda T Shimony JS Coalson RS Lieu JEC. Diffusion tensor imaging in children with unilateral hearing loss: a pilot study. Front Syst Neurosci. (2014) 8:87. 10.3389/fnsys.2014.00087 24904310 PMC4033270

[8] Klee TM Davis-Dansky E. A comparison of unilaterally hearing-impaired children and normal-hearing children on a battery of standardized language tests. Ear Hear. (1986) 7(1):27–37. 10.1097/00003446-198602000-00006 3949098

[9] Masuda S Usui S. Comparison of the prevalence and features of inner ear malformations in congenital unilateral and bilateral hearing loss. Int J Pediatr Otorhinolaryngol. (2019) 125:92–7. 10.1016/j.ijporl.2019.06.028 31276892

[10] Paul A Marlin S Parodi M Rouillon I Guerlain J Pingault V Unilateral sensorineural hearing loss: medical context and etiology. Audiol Neurootol. (2017) 22(2):83–8. 10.1159/000474928 28738350

[11] Cone-Wesson B Vohr BR Sininger YS Widen JE Folsom RC Gorga MP Identification of neonatal hearing impairment: infants with hearing loss. Ear Hear. (2000) 21(5):488–507. 10.1097/00003446-200010000-00012 11059706

[12] Masuda S Usui S Matsunaga T. High prevalence of inner-ear and/or internal auditory canal malformations in children with unilateral sensorineural hearing loss. Int J Pediatr Otorhinolaryngol. (2013) 77(2):228–32. 10.1016/j.ijporl.2012.11.001 23200870

[13] Schiariti V Klassen AF Houbé JS Synnes A Lisonkova S Lee SK. Perinatal characteristics and parents’ perspective of health status of NICU graduates born at term. J Perinatol. (2008) 28(5):368–76. 10.1038/jp.2008.9 18288117

[14] Saigal S Doyle LW. An overview of mortality and sequelae of preterm birth from infancy to adulthood. Lancet. (2008) 371(9608):261–9. 10.1016/S0140-6736(08)60136-1 18207020

[15] Newnam K Parrott J. The NICU graduate: implications for pediatric primary care. Newborn Infant Nurs Rev. (2013) 13(2):94–100. 10.1053/j.nainr.2013.03.005

[16] Ministry of Housing CaLG. English indices of deprivation 2019 Postcode Lookup 2019. Available from: http://imd-by-postcode.opendatacommunities.org/imd/2019.

[17] Ministry of Housing CaLG. The English Indices of Deprivation 2019 Frequently Asked Questions (FAQs) 2019. Available from: https://assets.publishing.service.gov.uk/government/uploads/system/uploads/attachment_data/file/853811/IoD2019_FAQ_v4.pdf.

[18] Audiology BSo. Pure-tone air-conduction and boneconduction threshold audiometry with and without masking. (2018) updated August 2018. Available from: https://www.thebsa.org.uk/wp-content/uploads/2018/11/OD104-32-Recommended-Procedure-Pure-Tone-Audiometry-August-2018-FINAL.pdf

[19] Strömland K Miller M Sjögreen L Johansson M Joelsson B-ME Billstedt E Oculo-auriculo-vertebral spectrum: associated anomalies, functional deficits and possible developmental risk factors. Am J Med Genet A. (2007) 143A(12):1317–25. 10.1002/ajmg.a.31769 17506093

[20] Arcand P Desrosiers M Dubé J Abela A. The large vestibular aqueduct syndrome and sensorineural hearing loss in the pediatric population. J Otolaryngol. (1991) 20(4):247–50. PMID: 1920576

[21] McGaughran JM Kuna P Das V. Audiological abnormalities in the Klippel-Feil syndrome. Arch Dis Child. (1998) 79(4):352. 10.1136/adc.79.4.352 9875048 PMC1717726

[22] Schick B Brors D Prescher A Draf W. Conductive hearing loss in Beckwith–Wiedemann syndrome. Int J Pediatr Otorhinolaryngol. (1999) 48(2):175–9. 10.1016/S0165-5876(99)00015-4 10375044

[23] Haffey T Fowler N Anne S. Evaluation of unilateral sensorineural hearing loss in the pediatric patient. Int J Pediatr Otorhinolaryngol. (2013) 77(6):955–8. 10.1016/j.ijporl.2013.03.015 23582232

[24] Friedman AB Guillory R Ramakrishnaiah RH Frank R Gluth MB Richter GT Risk analysis of unilateral severe-to-profound sensorineural hearing loss in children. Int J Pediatr Otorhinolaryngol. (2013) 77(7):1128–31. 10.1016/j.ijporl.2013.04.016 23701899

[25] Niu K Brandström A Skenbäck S Duan M Uhlén I. Risk factors and etiology of childhood hearing loss: a cohort review of 296 subjects. Acta Otolaryngol. (2020) 140(8):660–6. 10.1080/00016489.2020.1757753 32401111

[26] Tomblin JB Harrison M Ambrose SE Walker EA Oleson JJ Moeller MP. Language outcomes in young children with mild to severe hearing loss. Ear Hear. (2015) 36(Suppl 1(0 1)):76S–91S. 10.1097/AUD.0000000000000219 26731161 PMC4704115

[27] Krishnan LA Van Hyfte S. Management of unilateral hearing loss. Int J Pediatr Otorhinolaryngol. (2016) 88:63–73. 10.1016/j.ijporl.2016.06.048 27497389

[28] McKay S. To aid or not to aid: children with unilateral hearing loss Audiology Online [updated 07/22/2002]. Available from: https://www.audiologyonline.com/articles/to-aid-or-not-children-1167

[29] Briggs L Davidson L Lieu J. Outcomes of conventional amplification for pediatric unilateral hearing loss. Ann Otol Rhinol Laryngol. (2011) 120:448–54. 10.1177/000348941112000705 21859053 PMC3469201

[30] Banga R Doshi J Child A Pendleton E Reid A McDermott AL. Bone-anchored hearing devices in children with unilateral conductive hearing loss: a patient-carer perspective. Ann Otol Rhinol Laryngol. (2013) 122(9):582–7. 10.1177/000348941312200908 24224402

[31] Brown KD Dillon MT Park LR. Benefits of cochlear implantation in childhood unilateral hearing loss (CUHL trial). Laryngoscope. (2022) 132(Suppl 6(Suppl 6)):S1–S18. 10.1002/lary.29853 34542181 PMC9293149

[32] England PH. Patient Journey from screen to referral GOV UK2020 [updated 14/12/2020]. Available from: https://www.gov.uk/government/publications/newborn-hearing-screening-programme-nhsp-operational-guidance/6-patient-journey-from-screen-to-referral

[33] England PH. Newborn hearing screening programme (NHSP): care pathways for babies in neonatal intensive care units (NICU) GOV UK2020 [updated 29/10/2020]. Available from: https://www.gov.uk/government/publications/newborn-hearing-screening-care-pathways/newborn-hearing-screening-programme-nhsp-care-pathways-for-babies-in-neonatal-intensive-care-units-nicu

[34] England PH. Newborn hearing screening programme (NHSP): care pathways for well babies GOV UK2020 [updated 29/10/2020]. Available from: https://www.gov.uk/government/publications/newborn-hearing-screening-care-pathways/newborn-hearing-screening-programme-nhsp-care-pathways-for-well-babies

[35] England PH. Guidelines for surveillance and audiological referral for infants and children following newborn hearing screen GOV UK2019 [updated 19/07/2019]. Available from: https://www.gov.uk/government/publications/surveillance-and-audiological-referral-guidelines/guidelines-for-surveillance-and-audiological-referral-for-infants-and-children-following-newborn-hearing-screen

[36] Approach to the Child with Birth Defects http://www.med.umich.edu/lrc/coursepages/m1/humangenetics/HG501-Petty-Lec16.html.

