# Supplementary material for: Multiple congenital anomalies and adverse developmental outcomes are associated with neonatal intensive care admission and unilateral hearing loss
Source: Front Pediatr. 2023 Jan 10;10:1068884. doi: 10.3389/fped.2022.1068884 (PMC9873408; doi:10.3389/fped.2022.1068884)
Supplement: Supplementary file 1 [file Datasheet1.docx]

**Supplementary Materials**

**Appendix 1 - GOV UK Guidelines for Detection of UHL**

Babies that have been admitted to the NICU for >48hrs undergo both Automated Auditory Brainstem Response (AABR) and Automated Otoacoustic Emission (AOAE) testing (31, 32). If no clear response or an inconclusive response is detected in one or both ears with the AABR test, then the child is referred to diagnostic services for an immediate audiology assessment (within 4 weeks of screening or by 44 weeks gestational age) (32). Follow up audiological assessment is also required in 7 to 9 months if these babies receive a clear response on AABR but no clear response on AOAE in both ears (32).

Well babies (those that have not been admitted to the NICU or spend <48hrs there) only undergo AABR testing if they don’t have a clear response in both ears in the first two AOAE tests (33). If there is not a clear response in both ears for the AOAE tests *and* AABR test then the baby is referred for immediate audiological assessment (33).

There are four main contraindications to screening, which are microtia or atresia of the external ear canal, bacterial meningitis, CMV and a programmable ventriculo-peritoneal shunt; in all of these cases, the child should be referred for immediate audiological assessment instead of undergoing screening (34).

**Appendix 2 – Specific Diagnoses Included in Each Sub-Group of Abnormal Anatomical Features**

Diagnoses in *italics* were identified by a consultant neonatologist as being congenital.

Inner ear malformations

- *Cochlear nerve aplasia*
- *Absent auditory nerve*
- *Enlarged vestibular aqueduct*
- *Narrow ear canal*
- *Eustachian tube dysfunction*
- *Vestibulocochlear nerve palsy*
- *Meatal atresia*

Craniofacial abnormalities

Minor - an anomaly that was considered a variation of the normal spectrum and did not significantly affect the child’s health (35).

- *Pre-auricular skin tag*
- *Cyst on ear*
- *Cheek skin tag*
- *Cryptotia*
- *Plagiocephaly*
- *Dysmorphic facial features*
- *Creases in ear lobes*
- *Small close set eyes*
- *Small mouth*
- *Facial asymmetry*
- *Abnormal head shape*
- *Cyst behind ear*
- *Short and upslanting palpebral fissures*
- *Posteriorly rotated ears*
- *Retrognathia*
- *Small chin*

Major - an anomaly that was not a variation of the normal spectrum and significantly affected the child’s health (34)

- *Malformation of ear*
- *Unilateral cleft lip*
- *Microtia*
- *Facial nerve palsy*
- *Cyst back of skull*

Neurological and spinal

- *Craniosynostosis*
- Periventricular flare
- Seizure
- Encephalitis
- Hydrocephalus
- *Wide sagittal suture*
- *Large anterior fontanelle*
- *Choroid plexus cysts*
- *Sacral hemivertebrae*
- *Abnormal cervical vertebral bodies*
- *Butterfly vertebrae*

Gastrointestinal

- *Oesophageal atresia*
- *Tracheoesophageal fistula*
- *Oesophageal stricture*
- *Inguinal hernia*
- Umbilical hernia
- Necrotising enterocolitis (GI)

Vision and eye

- Nystagmus
- Vision impairment
- Long sighted
- Astigmatism
- *Esotropia*
- *Microtropia*
- Chalazion
- Retinopathy of prematurity

Other malformations

- *Sacral dimple*
- *Hypospadias*
- *Equinovalgus malformation*
- *Single umbilical artery*
- *Dysplastic hip*

Cardiac

- *Ventricular septal defect*
- *Atrial septal defect*
- *Patent foramen ovale*
- *Patent ductus arteriosus*
- *Tetralogy of fallot*

Respiratory:

- *Congenital lobar emphysema*
- Chronic lung disease
- Bronchopulmonary dysplasia
- *Small upper airway/ Laryngomalacia*
- *Tracheal stenosis*
- Meconium aspiration syndrome
- Congenital pneumonia

Metabolic:

- Neonatal hypoglycaemia
- Hyperinsulinism
- *Hypothyroidism*
- *Pituitary cyst*
- Congenital lactic acidosis

Renal:

- *Small kidney*
- *Vesico-uteric reflux*
- Neonatal acute renal failure
- *Renal cyst*
- *Lobulated and enlarged kidney*

Neuromotor:

- Increased tone in limbs
- *Hypermobility*
- Frequent trips and falls
- Motor co-ordination difficulties
- Mobility issues
- Head lag
- Hypotonia

Speech and language therapy – it was recorded in the patient notes that they had received SLT in their childhood.

Developmental impairment – it was stated in the patient notes that they had a global developmental delay, fine motor delay, delay in communication, developmental impairment or had been referred for developmental needs.

Learning disability – had a diagnosed learning disability.

Autism – had a diagnosis of Autism.
